# Diversity of 3′ variable region of *cagA* gene in *Helicobacter pylori* strains isolated from Chinese population

**DOI:** 10.1186/s13099-021-00419-3

**Published:** 2021-04-13

**Authors:** Zhijing Xue, Yuanhai You, Lihua He, Yanan Gong, Lu Sun, Xiurui Han, Ruyue Fan, Kangle Zhai, Yaming Yang, Maojun Zhang, Xiaomei Yan, Jianzhong Zhang

**Affiliations:** State Key Laboratory of Infectious Disease Prevention and Control, Collaborative Innovation Center for Diagnosis and Treatment of Infectious Diseases, Chinese Center for Disease Control and Prevention, National Institute for Communicable Disease Control and Prevention, Beijing, China

**Keywords:** *Helicobacter pylori*, *cagA*, EPIYA, Gastroduodenal disease, Polymorphism

## Abstract

**Background:**

The cytotoxin-associated gene A (*cagA*) is one of the most important virulence factors of *Helicobacter pylori* (*H. pylori*). There is a highly polymorphic Glu-Pro-Ile-Tyr-Ala (EPIYA) repeat region in the C-terminal of CagA protein. This repeat region is thought to play an important role in the pathogenesis of gastrointestinal diseases. The aim of this study was to investigate the diversity of *cagA* 3′ variable region and the amino acid polymorphisms in the EPIYA segments of the CagA C-terminal region of *H. pylori*, and their association with gastroduodenal diseases.

**Methods:**

A total of 515 *H. pylori* strains from patients in 14 different geographical regions of China were collected. The genomic DNA from each strain was extracted and the *cagA* 3′ variable region was amplified by polymerase chain reaction (PCR). The PCR products were sequenced and analyzed using MEGA 7.0 software.

**Results:**

A total of 503 (97.7%) *H. pylori* strains were *cagA*-positive and 1,587 EPIYA motifs were identified, including 12 types of EPIYA or EPIYA-like sequences. In addition to the four reported major segments, several rare segments (e.g., B′, B″ and D′) were defined and 20 different sequence types (e.g., ABD, ABC) were found in our study. A total of 481 (95.6%) strains carried the East Asian type CagA, and the ABD subtypes were most prevalent (82.1%). Only 22 strains carried the Western type CagA, which included AC, ABC, ABCC and ABCCCC subtypes. The CagA-ABD subtype had statistical difference in different geographical regions (P = 0.006). There were seven amino acid polymorphisms in the sequences surrounding the EPIYA motifs, among which amino acids 893 and 894 had a statistical difference with gastric cancer (P = 0.004).

**Conclusions:**

In this study, 503 CagA sequences were studied and analyzed in depth. In Chinese population, most *H. pylori* strains were of the CagA-ABD subtype and its presence was associated with gastroduodenal diseases. Amino acid polymorphisms at residues 893 and 894 flanking the EPIYA motifs had a statistically significant association with gastric cancer.

## Background

*Helicobacter pylori* (*H. pylori*) is a spiral, microaerophilic Gram-negative bacterium that colonizes the gastric mucosa of more than half of the world’s population [2]. *H. pylori* infection is not only closely related to chronic gastritis (CG) and peptic ulcer disease (PUD) but also an important risk factor for gastric adenocarcinoma and mucosal-associated lymphoid tissue (MALT) lymphoma. Therefore, the World Health Organization classified *H. pylori* as a group I carcinogen in 1994 [34]. Epidemiological survey shows that about 50% of adults are infected with *H. pylori* and chronic infection of *H. pylori* plays an important role in the development of gastric carcinoma [24]. Despite the high prevalence of *H. pylori* infection, more than 80% of the carriers present asymptomatic gastritis, only 10%–20% develop CG and PUD, and a minority of *H. pylori* carriers develop into gastric cancer (GC) [22]. Variation in virulence of the strains is thought to be an important reason for the different clinical outcomes of *H. pylori* infection [43]. The cytotoxin-associated gene A (*cagA*) is one of the most important virulence genes of *H. pylori*, which is located at the end of *cag* pathogenicity island (*cag* PAI) and encodes the 120–145 kDa CagA protein [7]. Studies have confirmed that the *cagA*-positive strains are more virulent than the *cagA*-negative strains and can cause more severe gastric inflammation [5]. CagA protein can be transported into the gastric epithelial cells by type IV secretion system (T4SS) encoded by the *cag* PAI. After the CagA translocation, the tyrosine residues of EPIYA(Glu-Pro-Ile-Tyr-Ala) motif in the CagA C-terminal region can be phosphorylated by Src family kinases (SFKs) rapidly [21, 30]. Based on the amino acid sequences flanking the EPIYA motifs, the EPIYA motifs can be subdivided into four distinct peptide segments: EPIYA-A, EPIYA-B, EPIYA-C and EPIYA-D [23]. According to the different combinations of these four EPIYA motifs, *H. pylori* can be divided into two types, namely the East Asian type and the Western type [15].

CagA can specifically bind to the SH2 domain of Src homology 2 (SH2)—containing protein tyrosine phosphatase (SHP-2), which induces spatial configuration change of SHP-2 and activates it [40]. SHP-2 can be involved in the downstream signal transduction of growth factor receptor, regulate cell growth, differentiation and cell adhesion, and thereby inducing morphologic transformation and abnormal proliferation of gastric epithelial cells [6]. The binding of CagA and SHP-2 can lead to the cytoskeletal rearrangement of the host gastric epithelial cells, known as the hummingbird phenotype, which plays an important role in the development of gastric cancer [14]. Studies showed that the East Asian type CagA containing EPIYA-D segment displayed stronger binding activity to SHP-2 and more strongly damage to cells than did Western CagA. Western strains with more EPIYA-C segments showed a stronger ability to bind to SHP2 and could be prone to induce the hummingbird phenotype than Western type CagA containing segments EPIYA-C [9]. The phosphorylated CagA can interact with CagA C-terminal Src kinase (Csk) and inactivate Src kinase, resulting in cytoskeleton rearrangement and cell elongation [13]. In addition to SHP-2 and Csk, the CagA protein can interact with PI3K (phosphatidylinositol 3-kinase), Grb2 (growth factor receptor bound protein 2) and ZO-1 (zonula occludens-1) in a tyrosine phosphorylation-dependent manner [1, 18]. The interaction of CagA with these proteins results in activation of abnormal signaling pathways that can lead to cell dysfunction. In addition, the CagA C-terminus includes a 16 amino acid stretch named CagA-multimerization (CM) sequence or conserved repeat responsible for phosphorylation-independent activity (CRPIA) motif located distal to the EPIYA-C or EPIYA-D segment [36]. The CRPIA motif can mediate the dimer formation of CagA protein and stabilize its binding to SHP-2 [25]. It can also bind to polarity regulatory kinase partitioning defective 1/microtubule affinity regulating kinase (PAR1/MARK) and inhibit the activity of kinase [29].

The incidence of *H. pylori* infection and gastric cancer in China is much higher than that in the Western countries [35]. However, there are controversial reports about the relationship between the CagA type and gastroduodenal diseases [28, 44]. This controversy may be due to regional diversity or differences in research methods. In fact, there is lack of comprehensive analysis of *cagA* 3′ variable region sequence characteristics. Moreover, few studies have detected the detailed amino acid polymorphisms surrounding the EPIYA motifs and their association with clinical outcomes [3]. The aim of this study was to investigate the diversity of *cagA* 3′ variable region and the amino acid polymorphisms surrounding the EPIYA motifs, and the relationship with gastroduodenal diseases through the sequence alignment and statistical analysis of 503 CagAs in *H. pylori* strains isolated from Chinese different populations.

## Results

### *cagA* gene status

**Fig. 1:**
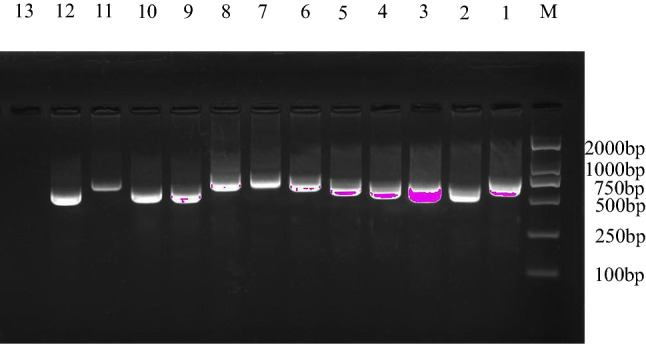
Polymerase chain reaction (PCR) products of the *cagA* 3′ variable region. Lane 1, subtype ABBD; Lane 2, subtype ABBB; Lane 3, subtype AAABD; Lane 4, subtype ABBBD; Lane 5, subtype ABDABD; Lane 6, subtype AB′B′B′B′BD; Lane 7, subtype AB′B′B′B′B′BD; Lane 8, subtype AB′BB″DAB′; Lane 9, subtype ABD; Lane 10, subtype ABCC; Lane 11, subtype ABCCCC; Lane 12, subtype ABC; Lane 13, negative control; M: DL2000 DNA marker

A total of 503 (97.7%) *cagA*-positive strains out of 515 *H. pylori* strains from 14 different geographical regions in China were obtained. Among those *cagA*-positive strains, 82 (91.1%) were isolated from Shandong, 75 (94.9%) from Guangxi and 100% from other twelve regions. There was no significant difference in the distribution among different regions (χ^2^ = 0.933, P > 0.05). The sequencing results showed that PCR products of the *cagA* 3′ variable region ranged from 480 to 858 bp, most of which were approximately 630 bp (Fig. 1). The alignment of the corresponding amino acid sequences revealed the presence of amino acid mutations, such as substitution, insertion and deletion in the CagA C-terminal region.

### Characteristics of EPIYA segments flanking sequences

**Fig. 2:**
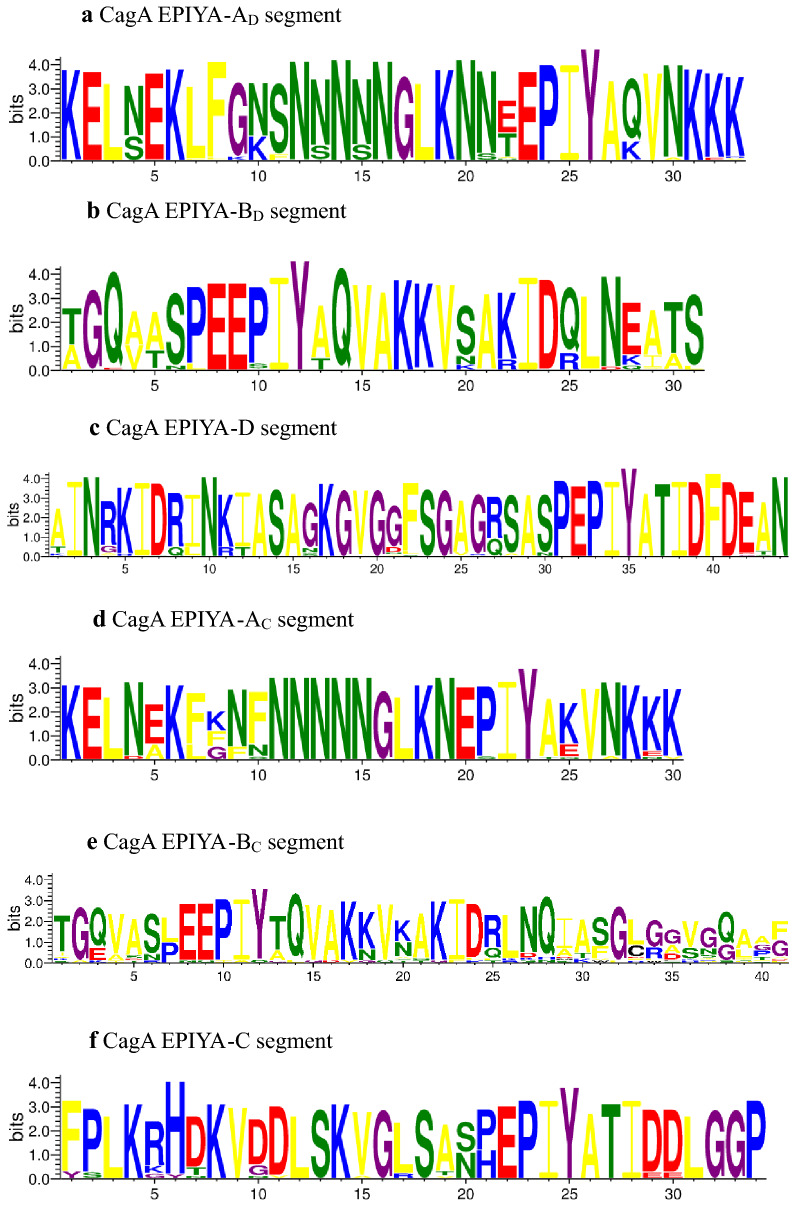
Variation in the EPIYA segment sequences of CagA protein

**Fig. 3:**
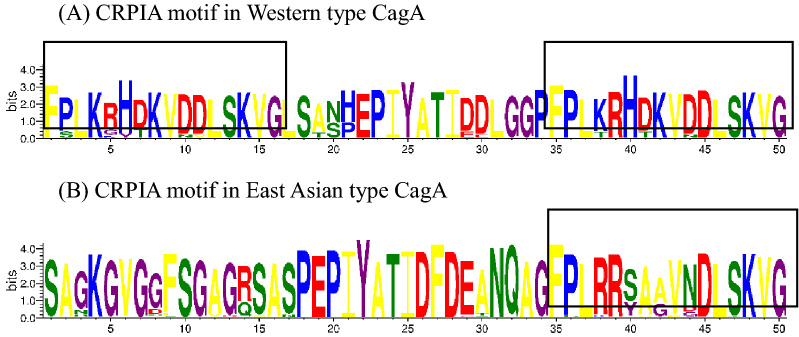
The CRPIA motifs in Western type and East Asian type CagA from Chinese strains. The target CRPIA motifs in EPIYA segments C and D are shown in frame

According to the segments flanking EPIYA motifs, we classified EPIYA segments. In addition to the four major segments, we defined several rare segments, including EPIYA-B′, EPIYA-B″ and EPIYA-D′. Representative segment types obtained from 503 CagAs were listed in Table 1. Through sequence alignment, it was found that there were differences in amino acids among the same sequences. The two most frequent segments in segments A, B, C and D were shown in Table 2. There were obvious differences between segments EPIYA-C and EPIYA-D when analyzed using the WebLogo 3. As shown in Fig. 2, the lengths and sequences of segments A_C_ and A_D_ were very similar, whereas that of segments B_C_ and B_D_ were quite different. The sequences variation started from the six amino acids, QVAKKV, in segments B_C_ and B_D_, and the sequences of segments C and D were completely different. As shown in Fig. 3, the most common CRPIA motif in Western type CagA was FPLKRHDKVDDLSKVG and the most common CRPIA motif in East Asian type CagA was FPLRRSAAVNDLSKVG. Western and East Asian CRPIA motifs varied at positions 4, 6, 7, 8 and 10. Western strains with three EPIYA motifs contained two CRPIA motifs and the CRPIA motif before the EPIYA-C motif was a Western type in all 22 Western type strains.

**Table 1:** Representative sequences of EPIYA repeat region

Segment	No.	Representative sequences
A_D_	483	KELNEKLFGNSNNNNNGLKNNT**EPIYA**QVNKKK
B_D_	478	TGQVASPE**EPIYA**QVAKKVSAKIDQLNEATS
B′_D_	56	TGQVASPE**EPIYA**QVNKKK
B″_D_	18	AINRKIDRINKIASAGKGVGGFSGAGQSASPE**EPIYA**QVAKKVSAKIDQLNESAS
D	468	AINRKIDRINKIASAGKGVGGFSGAGRSASP**EPIYA**TIDFDEAN
D′	12	FPLKRHDKVGDLSKVGLSASP**EPIYA**TIDFDEAN
A_C_	22	KELNEKFKNFNNNNNGLKN**EPIYA**KVNKKK
B_C_	22	TGQVASPE**EPIYA**QVAKKVNAKIDRLNQIASGLGGVGQAAG
C	28	FPLKRHDKVDDLSKVGLSASP**EPIYA**TIDDLGGP

The subscripts C and D indicate that sequences containing segments A, B, B′ and B″ contain segments C and D, respectively

**Table 2:** Two most frequent segments in EPIYA repeat region

Segment	Sequences	Ratio
A_D_	KELNEKLFGNSNNNNNGLKNNT**EPIYAQ**VNKKK	145/483
A_D_	KELNEKLFGNSNNNNNGLKNNT**EPIYAK**VNKKK	29/483
B_D_	TGQ**VA**SPE**EPIYA**QVAKKVSAKIDQLNEATS	98/478
B_D_	TGQ**AT**SPE**EPIYA**QVAKKVSAKIDQLNEATS	89/478
D	AINRKIDRINKIASAGKGVGGFSGAG**R**SASP**EPIYA**TIDFDEAN	179/468
D	AINRKIDRINKIASAGKGVGGFSGAG**Q**SASP**EPIYA**TIDFDEAN	85/468
A_C_	KELN**E**K**FK**NFNNNNNGLKN**EPIYA**KVNKKK	15/22
A_C_	KELN**A**K**LG**NFNNNNNGLKN**EPIYA**KVNKKK	6/22
B_C_	TGQ**V**ASPE**EPIYA**QVAKKVNAKIDRLNQIASGLGGVGQAAG	5/22
B_C_	TGQ**A**ASPE**EPIYA**QVAKKVNAKIDRLNQIASGLGGVGQAAG	2/22
C	FPLKRHDKVDDLSKVG**L**SASP**EPIYA**TIDDLGGP	16/28
C	FPLKRHDKVDDLSKVG**R**SVSP**EPIYA**TIDDLGGP	4/28

Different amino acids in the two sequences are highlighted; Ratio = (Number of the segment)/(Total)

The alignment of the amino acid sequences confirmed that the EPIYA motifs in the EPIYA-C and EPIYA-D segments were highly conserved, whereas the EPIYA motifs in the EPIYA-A and EPIYA-B segments had evidence of mutations. A total of 1,587 EPIYA motifs were obtained from the 503 CagAs, including 12 types of EPIYA or EPIYA-like sequences (Table 3). The three most frequent EPIYA motifs were EPIYA (1461/1587 = 92.1%), EPIYT (4.7%), and ESIYA (1.4%). The EPIYA-B motif had a high degree of variation in the five amino acids (e.g., EPIYA, EPIYT and ESIYA). As shown in Table 4 and Fig. 2, the sequences, KVNK and QVNK, were the main types of segments A_C_ and A_D_, respectively. QVAK was the main amino acid of segments B_C_ and B_D_. In the present study, the sequences were identified as segments C and D if they were followed by TIDD and TIDF, respectively. However, by sequence alignment, it also belonged to segment C if it was followed by TIED or TIDE.

**Table 3:** Distribution of EPIYA motifs in segments A, B, C and D

Type	Distribution	Total
A	502 EPIYA, 1 ESIYA, 1 EPVYA, 1 EPIYT	505
B, B′, B″	450 EPIYA, 73 EPIYT, 22 ESIYA, 9 EPLYA, 7 ESIYT, 5 ELIYA, 3 EHIYA, 1 EAIYA, 1 APIYA, 1 ELIYA, 1 DPIYA	573
D, D′	480 EPIYA	480
C	29 EPIYA	29
Total		1587

**Table 4:** Distribution of the first four amino acids following EPIYA motifs

Type	EPIYA-D		EPIYA-C	Total
	Occurrence and short segment	Total	Occurrence and short segment	
A	356 QVNK, 119 KVNK, 4 EVNK, 4 QVAK	483	16 KVNK, 6 EVNK	22
B	477 QVAK, 60 QVNK, 4 KVNK, 3 QIAK, 2 QVTK, 2 QVAR, 1 QLTK, 1 QITK, 1 QVAQ, 1 QVNG	552	22 QVAK	22
C/D	480 TIDF	480	24 TIDD, 2 TIDE, 2 TIED	28

### CagA sequence types classification

A total of 20 sequence types were obtained from 503 CagAs (Table 5). CagA type was mainly East Asian type, accounting for 95.6% (481/503). The majority of the sequences were of types ABD (82.1%, 413/503) and AB′BD (8.2%, 41/503). There were only 22 strains of Western type, including types AC, ABC, ABCC and ABCCCC. There were 1–8 EPIYA motifs in CagA C-terminal region, and 87.3% (439/503) of the strain sequences had three EPIYA segments. The sequences containing 1 through 8 EPIYA segments were 1, 6, 439, 46, 6, 2, 2 and 1, respectively. For example, there was only one EPIYA segment D in the sequence of type D and eight EPIYA segments in the sequence of AB′B′B′B′B′BD, including six repeats of segment B.

**Table 5:** Number of the sequence types

Seq. Type	No.	Seq. Type	No.	Seq. Type	No.	Seq. Type	No.
ABD	413	AAABD	1	BD	1	D	1
ABD′	5	ABDABD	1	AB′BB″DAB′	1	ABC	16
AB-D′	5	AD	2	AB′B′B′B′BD	1	ABCC	4
AB′BD	41	AD′	1	ABB″B″	1	ABCCCC	1
AB′B′BD	5	A-D′	1	AB′B′B′B′B′BD	1	AC	1

The hyphen indicates that there is no EPIYA motif between two adjacent EPIYA segments

### Correlation between CagA sequence types and geographical regions

There were some differences in CagA sequence types in different geographical regions (Table 6). In Yunnan, strains containing 4 or more EPIYA motifs accounted for 40% (29/73). There was a significant correlation between CagA-AB′BD type and Yunnan isolates (χ^2^ = 81.523, P < 0.001). However, most of the Western strains were from Neimenggu, and the association of CagA-ABC type with Neimenggu isolates was highly significant (χ^2^ = 25.468, P < 0.01). There was a significant difference in the presence of CagA-ABD type between different geographical regions (χ^2^ = 80.067, P < 0.01).

**Table 6:** The distribution of CagA sequence types in different geographical regions

CagA type		Geographical regions														Total
		FJ	SD	GX	YN	HL	HN	NM	QH	ZJ	BJ	NX	TW	SX	XZ	
East Asian type CagA	ABD^*^	65	75	69	41	49	46	18	32	6	4	3	4	1	0	413
	ABD′	0	1	3	0	0	0	1	0	0	0	0	0	0	0	5
	AB-D′	0	0	0	0	0	0	5	0	0	0	0	0	0	0	5
	AB′BD	10	3	1	24^†^	1	0	0	0	0	0	1	0	0	1	41
	AB′B′BD	2	0	0	3	0	0	0	0	0	0	0	0	0	0	5
	AAABD	1	0	0	0	0	0	0	0	0	0	0	0	0	0	1
	ABDABD	1	0	0	0	0	0	0	0	0	0	0	0	0	0	1
	AD	0	0	0	0	1	0	1	0	0	0	0	0	0	0	2
	AD′	1	0	0	0	0	0	0	0	0	0	0	0	0	0	1
	A–D′	0	0	0	0	0	0	0	0	0	0	0	0	1	0	1
	BD	0	1	0	0	0	0	0	0	0	0	0	0	0	0	1
	AB′BB″DAB′	1	0	0	0	0	0	0	0	0	0	0	0	0	0	1
	AB′B′B′B′BD	0	0	0	1	0	0	0	0	0	0	0	0	0	0	1
	ABB″B″	1	0	0	0	0	0	0	0	0	0	0	0	0	0	1
	AB′B′B′B′B′BD	0	0	0	0	0	0	0	0	1	0	0	0	0	0	1
	D	1	0	0	0	0	0	0	0	0	0	0	0	0	0	1
Total		83	80	73	69	51	46	25	32	7	4	4	4	2	1	481
western type CagA	ABC	0	2	1	2	5	0	5^‡^	1	0	0	0	0	0	0	16
	ABCC	0	0	1	1	0	0	2	0	0	0	0	0	0	0	4
	ABCCCC	0	0	0	0	0	0	1	0	0	0	0	0	0	0	1
	AC	0	0	0	1	0	0	0	0	0	0	0	0	0	0	1
Total		0	2	2	4	5	0	8	1	0	0	0	0	0	0	22
All CagA type total		83	82	75	73	56	46	33	33	7	4	4	4	2	1	503

Isolates were from 14 regions: Fujian (FJ), Shandong (SD), Guangxi (GX), Yunnan (YN), Heilongjiang (HL), Hunan (HN), Neimenggu (NM), Qinghai (QH), Zhejiang (ZJ), Beijing (BJ), Ningxia (NX), Taiwan (TW), Shanxi (SX) and Xizang (XZ). ^*^ There was a significant difference in the presence of CagA-ABD type between different geographical regions (P < 0.01). ^†^ CagA-AB′BD type was signifcantly associated with Yunnan isolates (P < 0.001). ^‡^ CagA-ABC type was signifcantly associated with Neimenggu isolates (P < 0.01)

### Correlation between CagA sequence types and clinical outcomes

Clinical data were available from 131 of the 503 *cagA*-positive *H. pylori* strains. Based on the gastrointestinal endoscopy and pathological examination, CG was diagnosed in 85 patients, GC in 22, gastric ulcer (GU) in 10, duodenal ulcer (DU) in 10 and MALT lymphoma in 4. A total of 12 Western type strains were found, 11 of which were from patients with CG. Among all the 131 CagAs, 86 were of the type ABD, 25 of the type AB′BD, 3 of the type AB′B′BD, 2 of the type AB′B′B′B′BD and 3 of the type AD. The distribution of the CagA sequence types in various clinical outcomes was shown in Table 7. We compared the types ABD and AB′BD in relation to clinical outcomes. Other CagA types were excluded because the number of other types was relatively small. As shown in Table 7, the prevalence of ABD was 58.1% (50/86) in CG; whereas only 22.1% (19/86) in GC and 9.3% (8/86) in GU. The ratio of AB′BD /ABD was therefore higher in CG (20/50 = 0.4) than GC (1/19 = 0.05), and the differences were statistically significant (χ^2^ = 71.500/80.067, P < 0.01).

**Table 7:** CagA sequence types and clinical outcomes

	ABD	AB′BD	AB′B′BD	AB′B′B′B′BD	AD	AC	ABC	ABCC	ABCCCC	Total (%)
CG	50^*^	20^†^	2	1	1	1	6	3	1	85 (64.9)
GC	19	1	0	1	1	0	0	0	0	22 (16.8)
GU	8	1	0	0	1	0	0	0	0	10 (7.6)
DU	5	3	1	0	0	0	1	0	0	10 (7.6)
MALT lymphoma	4	0	0	0	0	0	0	0	0	4 (3.1)
Total	86	25	3	2	3	1	7	3	1	131 (100)

^*^ The prevalence of CagA-ABD was signifcantly higher in CG than in GC (P < 0.01). ^†^ The prevalence of CagA-AB′BD was signifcantly higher in CG than in GC (P < 0.01)

### Amino acid polymorphisms flanking the EPIYA motifs

**Fig. 4:**
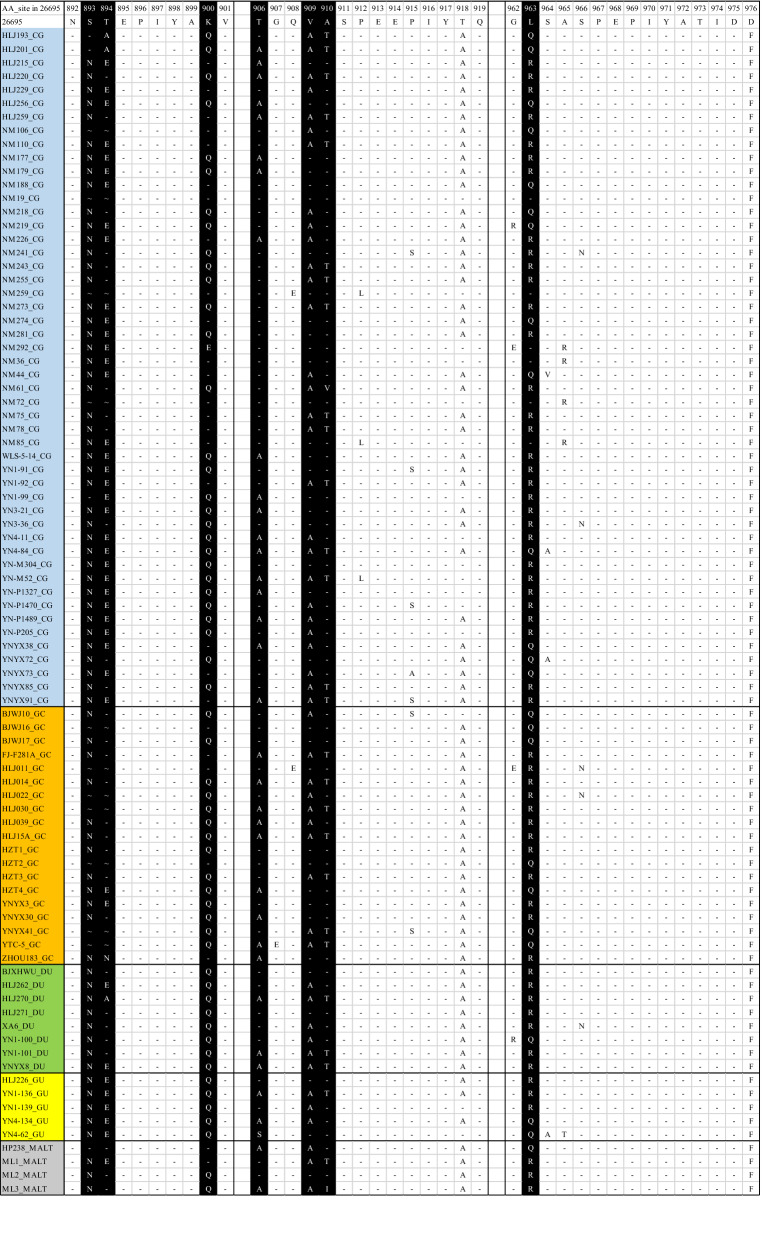
CagA amino acid comparison between CG, GC, GU, DU and MALT lymphoma strains. Seven amino acid polymorphisms are shown in white letters with black background. Identical amino acids are indicated by hyphens. Wavy lines indicate gap. The sequences start at position 892 of a reference CagA amino acid (strain 26695, CP003904)

Sequence alignment analysis showed that there were amino acid polymorphisms flanking the EPIYA motifs of *H. pylori* CagA C-terminal region. There were seven amino acid polymorphisms, at residues 893, 894, 900, 906, 909, 910 and 963, where the substitution rate of amino acids was more than 18.6% ([5 + 11]/ [70 + 5 + 11]). The detailed information of these amino acid polymorphisms in the sequence flanking the EPIYA motifs in 86 ABD subtypes was shown in Table 8 and Fig. 4. The absence of amino acids 893 and 894 was synchronous. Strains at the absence of the 893 and 894 residues had a statistically significant association with GC compared with CG (χ^2^ = 21.778, P < 0.01). Most patients with CG, GU, DU and MALT lymphoma had a glutamic acid (Glu) at 894, while some patients with GC had Glu deletion or substituted by other amino acids, such as threonine (Thr) or asparagine (Asn). These changes at residue 894 had significant difference between GC patients and those with other diseases (χ^2^ = 4.908, P < 0.05). In addition to the seven amino acid polymorphisms mentioned above, other amino acids were relatively conserved, except for individual amino acid absence or substitution.

**Table 8:** Amino acid polymorphisms in the sequence flanking the EPIYA motifs in 86 ABD subtypes

Disease	893			894				900			906		909		910			963			Total
	N	S	~	E	T	~	A/N	Q	K	E	T	A	A	V	A	T	V/I	R	Q	L	
CG	43 (86)	3 (6)	4 (8)	32 (64)	12 (24)	4 (8)	2 (4)	29 (58)	20 (40)	1 (2)	32 (64)	18 (36)	28 (56)	22 (44)	34 (68)	15 (30)	1 (2)	34 (68)	15 (30)	1 (2)	50
GC	12 (63.2)	0 (0)	7 (36.8) ^*^	2 (10.5) ^†^	9 (47.4)	7 (36.8) ^c^	1 (5.3)	14 (73.7)	5 (26.3)	0 (0)	10 (52.6)	9 (47.4)	10 (52.6)	9 (47.4)	12 (63.2)	7 (36.8)	0 (0)	12 (63.2)	7 (36.8)	0 (0)	19
GU	8 (100)	0 (0)	0 (0)	5 (62.5)	2 (25)	0 (0)	1 (12.5)	8 (100)	0 (0)	0 (0)	5 (62.5)	3 (37.5)	6 (75)	2 (25)	5 (62.5)	3 (37.5)	0 (0)	7 (87.5)	1 (12.5)	0 (0)	8
DU	4 (80)	1 (20)	0 (0)	4 (80)	0 (0)	0 (0)	1 (20)	5 (100)	0 (0)	0 (0)	2 (40)	3 (60)	3 (60)	2 (40)	4 (80)	1 (20)	0 (0)	3 (60)	2 (40)	0 (0)	5
MALT lymphoma	3 (75)	1 (25)	0 (0)	3 (75)	1 (25)	0 (0)	0 (0)	2 (50)	2 (50)	0 (0)	2 (50)	2 (50)	3 (75)	1 (25)	2 (50)	1 (25)	1 (25)	3 (75)	1 (25)	0 (0)	4
Total	70 (81.4)	5 (5.8)	11 (12.8)	46 (53.5)	24 (27.9)	11 (12.8)	5 (5.8)	58 (67.4)	27 (31.4)	1 (1.2)	51 (59.3)	35 (40.7)	50 (58.1)	36 (41.9)	57 (66.3)	27 (31.4)	2 (2.3)	59 (68.6)	26 (30.2)	1 (1.2)	86

Amino acid polymorphisms in the sequence flanking the EPIYA motifs in 86 ABD subtypes strains isolated from Chinese population. N, asparagine; S, serine; E, glutamic acid; T, threonine; A, alanine; Q, glutamine; K, lysine; V, valine; I, isoleucine; R, arginine; L, Leucine. Wavy lines indicate gap. Numbers in parentheses are percentages. ^*^ The absence of amino acids 893 and 894 was significantly associated with GC (P < 0.01). ^†^ The Glu deletion or substituted by other amino acids at residue 894, such as threonine (Thr) or asparagine (Asn), was significantly associated with GC (P < 0.05)

## Discussion

CagA is an important oncoprotein that can be translocated into the gastric epithelial cells and subsequently tyrosine-phosphorylated at residues of the EPIYA motifs [11]. The phosphorylated CagA can activate the phosphatase SHP-2 and then cause actin cytoskeleton rearrangement, hummingbird phenotype, which disturbs the normal signal transduction pathway of cells and promotes abnormal proliferation of gastric epithelial cells [6]. A recent study shows that the CagA EPIYA segments can interact with SHP-1, Grb2, Grb7, PI3K and Ras-GAP1 in addition to SHP-2 and Csk [32]. The interaction between CagA and these proteins suggests that CagA plays an important role in the development of gastrointestinal diseases caused by *H. pylori*. Therefore, we used molecular epidemiological methods to study the diversity of *cagA* 3′ variable region and the amino acid polymorphisms in the EPIYA segments of the CagA C-terminal region, and their association with gastroduodenal diseases.

The tyrosine phosphorylation site is located on EPIYA repeat sequences at the CagA C-terminus, and the number of EPIYA repeats directly affects the binding of CagA to SHP-2 and the ability of causing morphological changes of gastric epithelial cells [27]. Therefore, the variation of EPIYA repeat sequences may be an important reason for the difference in *H. pylori* strains virulence and clinical outcome. In our study, EPIYA (92.1%) was the predominant type, followed by EPIYT (4.7%) and ESIYA (1.4%). This result differed from previous study examining 710 EPIYA motifs of 206 CagAs, found 77.8% were EPIYA. EPIYT and ESIYT were only found in 14.8% and 6.2% of strains, respectively [17]. EPIYA motif variation had the highest frequency in EPIYA-B segment. It was reported that there was significant correlation between gastric cancer and EPIYA sequences, whereas EPIYT sequences was significantly associated with DU [42]. The role of EPIYT sequences in the development of gastrointestinal diseases needs further study. In EPIYA-C and EPIYA-D segments, the amino acids following EPIYA motif are generally TIDD and TIDF, respectively, which is an important structural domain of binding SHP-2. Our study confirmed that the EPIYA belonged to segment C if it was followed by TIED or TIDE. However, it has been proven that EPIYA was also identified as segment C if it was followed by TIEE, SIDD, TIDG, TIAE or TIAD, and it belonged to segment D if followed by TIDS [40].

According to the segments flanking the EPIYA motifs, we defined several segments, including B′_D_, B″_D_ and D′. The sequences of B′_D_, B″_D_ and D′ segments had some differences from those of B and D segments. For example, the sequences before EPIYA were similar to those of D segment in B″_D_ segment, whereas the sequences after EPIYA were similar to those of A_D_ segment in B′_D_ segment. It has been reported that the distribution of CagA EPIYA segments shows great geographical differences. The EPIYA-A and EPIYA-B segments appeared in almost all *cagA*-positive strains, whereas EPIYA-C and EPIYA-D segments were characteristic of Western and East Asian CagA strains, respectively [41]. As expected, 95.6% (481/503) of the CagA strains contained segment D. In contrast, 4.4% (22/503) contained segment C instead of segment D. Some studies showed that Western type CagA was the most frequent type in Mongolian and Russia patients and all *H. pylori* from gastric cancer patients possessed Western type CagA [26, 37]. Southeast Asian countries, such as Thailand and Myanma, formed the geographical boundaries between segments C and D, and the prevalence of CagA strains containing segments C and D was similar in Southeast Asian countries [20, 39]. In our study, 77.3% (17/22) of the Western CagAs were from Neimenggu, Heilongjiang and Yunnan, which may be due to human migration or direct transmission. Studies have reported that there was no significant correlation between CagA-ABD and the types of gastroduodenal diseases [44]. However, our study confirmed that there was a significant correlation between the ABD subtype and gastroduodenal diseases (P < 0.01). Studies have shown that East Asian CagA is more pathogenic than Western CagA, which may explain why the incidence of GC in Eastern countries is significantly higher than that in Western countries [19, 31]. In our study, the CRPIA motif of Western CagA showed approximately 70% identity with that of East Asian CagA. This result was consistent with previous studies [16]. Western CagA strains carried two CRPIA motifs, placed within each and distal to the ending of EPIYA-C, while a single CRPIA motif located after the EPIYA-D segment possesed by East Asian CagA.

CagA can be phosphorylated by the SFKs at tyrosine residues of the EPIYA motifs [30]. The tyrosine phosphorylated C and D segments specifically bind to SHP-2, which plays an important role in the development of gastric cancer [6]. The tyrosine phosphorylated A and B segments can bind and activate the CagA C-terminal Src kinase (CSK) that is a SFK with negative feedback regulation. The inhibition of SFK can lead to the decrease of phosphorylated CagA protein, which to some extent explains that *H. pylori* can survive in gastric epithelial cells for a long time without causing extensive gastric injury [8, 38]. Therefore, it is thought that CagA with more A and B segments can inhibit SFK more effectively, and thereby reduce cell damage [10]. In the present study, we found 20 CagA sequence types with different numbers of the EPIYA-A or EPIYA-B segment, such as AAABD, ABDABD and BD. The number of EPIYA-A and EPIYA-B segments may lead to the difference in the type and severity of gastrointestinal diseases. The relationship between EPIYA segments and gastrointestinal diseases needs to be further explored.

Research has shown that the pathogenicity of CagA is determined by the binding ability of SHP-2, which is also related to the number of tyrosine phosphorylation sites [6]. Souza [33] reported that the SH2 domains bound to highly correlated sequences, and the binding motif was pY-(S/T/A/V/I)-X-(V/I/L)-X-(W/F). Interestingly, the binding ability of East Asian CagA (pY-A-T-I-D-F) to SHP-2 was higher than that of Western CagA (pY-A-T-I-D-D), which can lead to more severe gastroduodenal diseases. Higashi et al. [12, 13] demonstrated that the difference of single amino acid led to the difference of SHP-2 binding activity between East Asian and Western CagA proteins. Therefore, the research on amino acid polymorphisms and their association with gastrointestinal diseases may have an important clinical value. In our study, we obtained seven amino acid polymorphisms in the sequences surrounding the EPIYA motifs: residues 893, 894, 900, 906, 909, 910 and 963. The absence of the amino acids 893 and 894 had a statistically significant association with GC. In most patients with CG, GU, DU and MALT lymphoma, the amino acids at residues 893 and 894 were asparagine (Asn) and glutamic acid (Glu), respectively, whereas 36.8% (7/19) of the isolates from GC patients lost these two amino acids. This change may affect the ability of CagA tyrosine phosphorylation and binding to SHP-2, and alter the spatial conformation of CagA protein, thereby accelerating the development of gastrointestinal diseases.

## Conclusions

In this study, 503 CagA sequences were analyzed in depth and we defined several novel segment types, including B′_D_, B″_D_ and D′. We demonstrated that most of *H. pylori* isolates from Chinese population were of the CagA-ABD subtype and it was statistically correlated with the type of gastroduodenal diseases. Strains at the absence or mutation of the 893 and 894 residues had a significant association with GC. Therefore, amino acid polymorphisms in EPIYA motifs might affect the function of CagA protein, and then lead to the development of gastrointestinal diseases, especially GC.

## Methods

### *H. pylori* culture and DNA extraction

A total of 515 *H. pylori* strains preserved in our laboratory were obtained from the following regions: Fujian (n = 83), Shandong (n = 90), Guangxi (n = 79), Yunnan (n = 73), Heilongjiang (n = 56), Hunan (n = 46), Neimenggu (n = 33), Qinghai (n = 33), Zhejiang (n = 7), Beijing (n = 4), Ningxia (n = 4), Taiwan (n = 4), Shanxi (n = 2), and Xizang (n = 1). *H. pylori* was streaked onto the Karmali agar plate supplemented with Karmali Agar base (CM 0935, Oxoid) containing 5% defibrinated sheep blood, and the plate was incubated at 37 °C for 3–5 days in a microaerobic atmosphere (5% O_2_, 10% CO_2_ and 85% N_2_). Bacteria were identified as *H. pylori* based on its external morphology, negative Gram staining and positive for catalase, oxidase and urease. The confirmed isolates were frozen at -80 °C until the genomic DNA was extracted with the QIAamp DNA Mini Kit (Qiagen, Germany) according to the manufacturer’s instructions. The extracted DNA was stored at -20℃ and used directly for PCR. This study was approved by Ethical Committee of National Institute for Communicable Disease Control and Prevention Chinese Center for Disease Control and Prevention (approval No. ICDC-2013001).

### PCR amplification

To amplify the *cagA* 3′ variable region of *H. pylori*, the primers were: forward, 5′-TGCGTGTGTGGCTGTTAGTAG-3′ and reverse, 5′-CCCTAGTCGGTAATGGGTTGT-3′ [4]. PCR assay was performed in a volume of 25 μl containing 1 μl of each primer, 1 μl template DNA, 12.5 μl Go Taq® Green Master Mix (Promega, USA) and 9.5 μl nuclease-free water. PCR was performed using a thermocycler system (Bio-Rad, USA) under the following conditions: denaturation at 94 °C for 5 min, 35 cycles at 94 °C for 30 s, at 54 °C for 30 s and at 72 °C for 40 s, and an extension at 72 °C for 10 min. The amplified products were identified after electrophoresis on 1.5% agarose gel with GelStain in 1 × TAE buffer at 110 V for 30 min. The gel documentation system (Bio-Rad, USA) was used to detect the DNA bands and obtain the images of the PCR products.

### Sequencing and analysis of the diversity of the cagA 3′ variable region

Positive PCR products were sent to the Beijing Genomics Institute (BGI) for purification and sequencing. The nucleotide sequences of the *cagA* 3′ variable region were submitted to China National Microbiological Data Center (accession number NMDCN0000IOV to NMDCN0000J0V and NMDCN0000LT9 to NMDCN0000M4U). EditPlus (version 5.3.0, korea) was used to collect sequence information, sort the sequences and create files in FASTA format. Bioedit was used to align and obtain amino acid sequences of the CagA protein. The EPIYA segment types and CRPIA motifs of CagA were analyzed using the program WebLogo (http://weblogo.threeplusone.com/). The Western strain 26695 *cagA* (GenBank No. CP003904) was used as a reference sequence. MEGA software (version 7.0.18, USA) was used for sequence alignments to analyze the diversity of the *cagA* 3′ variable region.

### Statistics

Statistical data were analyzed using SPSS 20.0 (SPSS, Chicago, USA). The χ^2^ test and Fisher’s exact test were used to test statistical difference among different gastroduodenal diseases in the CagA subtype and amino acid polymorphisms. A P-value < 0.05 was considered indicative of a statistically difference.

## Data Availability

The Western strain 26695 *cagA* was downloaded from NCBI (https://www.ncbi.nlm.nih.gov/).

## Abbreviations

*H. pylori*: *Helicobacter pylori*
PCR: Polymerase chain reaction
CG: Chronic gastritis
PUD: Peptic ulcer disease
GC: Gastric cancer
MALT: Mucosal-associated lymphoid tissue
GU: Gastric ulcer
DU: Duodenal ulcer
*cagA*: Cytotoxin-associated gene A
*cag* PAI: *cag* Pathogenicity island
T4SS: Type IV secretion system
SFKs: Src family kinases
SHP-2: Src homology 2 (SH2)—containing protein tyrosine phosphatase
CSK: CagA C-terminal Src kinase
CM: CagA-multimerization
CRPIA: Conserved repeat responsible for phosphorylation-independent activity
PAR1/MARK: Polarity regulatory kinase partitioning defective 1/microtubule affinity regulating kinase
